# A structured approach to effective mentoring of women health researchers in Africa

**DOI:** 10.4314/gmj.v56i3s.3

**Published:** 2022-09

**Authors:** Sylvie A Kwedi-Nolna, Marceline Djuidje-Ngounoue, Chanceline Bilounga-Ndongo, Mireille Ndje-Ndje, Elise S Mvodo-Meyo, Rose Leke

**Affiliations:** 1 Department of Public Health, Faculty of Medicine and Biomedical Sciences, University of Yaoundé 1, Cameroon; 2 Department of Biochemistry, Faculty of Science, University of Yaoundé 1, Cameroon; 3 Department of Psychology, Faculty of Arts, Lettres and Human Sciences, University of Yaoundé 1, Cameroon; 4 Department of Agricultural Economics and Agribusiness, Faculty of Agriculture and Veterinary Medicine, University of Buea, Cameroon; 5 Department for the Control of Disease, Epidemics and Pandemic, Ministry of Public Health, Yaoundé, Cameroon; 6 Department of Public Health, Faculty of Medicine and Pharmaceutical Sciences, University of Douala, Cameroon; 7 Higher Institute for Growth in Health Research for Women (HIGHER Women) Consortium; 8 The Biotechnology Centre, University of Yaoundé 1, Cameroon

**Keywords:** Mentoring, holistic approach, African-led research, capacity building, research sustainability

## Abstract

**Objectives:**

To formatively evaluate the HIGHER Women consortium's Mentor Protégée Program (MPP) and derive lessons for successful African women scientist mentorship.

**Design:**

Desk review of program documents and cross-sectional surveys of mentors and protégées.

**Setting:**

All 10 regions of Cameroon

**Participants:**

Women working in health research participating in the MPP.

**Interventions:**

Building health research skills and providing support for women to cope within the African psycho-social environment using a holistic approach.

**Main outcome measures:**

Formed mentor-protégés duos applying the MPP with measurable accomplishments.

**Results:**

The consortium counted 121 members with 103 protégées and 18 mentors. Of 103 protégées, 35 responded to the 2018 survey, while 77 responded to the 2022 survey. Mentioned benefits of the program included an increase in scientific peer-reviewed journal publications and presentations at national and international conferences. In the 2022 survey, a Pearson correlation showed an r of 0.41, which, although not statistically significant (p = .592), suggests a positive correlation between the increased number of peer-reviewed articles and increased number of years as HIGHER Women protégées.

**Conclusions:**

Mentorship programs can help over time to bridge the gender gaps within Africa as well as the gaps between African-led research and the rest of the world while making a meaningful contribution to enhancing the quality, diversity, and productivity of researchers. A mentoring program such as the HIGHER Women MPP can be improved by leveraging local and international partners to foster the mentoring program's sustainability, scalability, and expanded reach.

**Funding:**

World Health Organization's Special Programme for Research and Training in Tropical Diseases (WHO/TDR) and Canada's International Development Research Centre (IDRC Canada)

## Introduction

African women's contribution to high-quality published health research remains extremely low.^1^ This is due, among others, to obstacles and challenges such as insufficient national government support and other funding opportunities and an overwhelming academic and administrative workload, leaving no time for research.^2^

Men are three times more likely than women to reach top-level positions in the sciences and research.^3^ Mentoring strategies are effective in reducing this gender gap.^4^–^6^

To contribute to addressing some of these gender gaps, the “Higher Institute for Growth in Health Research for Women” (HIGHER Women) in Cameroon has put in place a Mentor-Protégée Program (MPP) to provide a structured approach to mentoring women researchers in Africa. This program is based on the premise that attracting and retaining more women in health research will maximise creativity and innovation and increase gender competency and expertise for optimal research output in Africa. The HIGHER Women's MMP mission is to support and encourage the sustainable professional growth of women scientists who are dedicated to improving the health of African communities through practice, service and research and aims to increase the number of women in low- and middle-income countries (LMICs) such as Cameroon doing health research as well as improve their access to research funding.^7^ The consortium believes that attracting and maintaining young female Africans in research is vital to developing African communities.^8^,^21^, ^22^,^23^

HIGHER Women was formed in 2015, with funding support from the World Health Organization's Special Programme for Research and Training in Tropical Diseases (WHO/TDR) and Canada's International Development Research Centre (IDRC), by Professor Rose Gana Fomban Leke, started her scientific career in the 1970s in Cameroon, and experienced at first hand the challenges of not having a mentor as a young woman in a profession dominated by men. As she journeyed to her present-day status as a world-acclaimed health researcher, she worked on changing this and providing mentoring opportunities to current and future generations of women scientists through HIGHER Women.

Since 2015, the consortium has brought together approximately 200 women comprising 170 protégées and 30 mentors. The positive effect of mentorship is supported by several theories, such as the Clutterbuck empirical model for mentorship, which portrays mentoring as essential for developing expertise and proficiencies through coaching.^9^ HIGHER women intentionally work to bring about change in the development of women's careers in health research by focusing on leadership, career promotion and overall growth.

This paper describes and analyses the HIGHER Women consortium's approaches, perceptions, experiences and lessons and outcomes of mentoring women entering careers in research for health in Cameroon and draws out lessons on factors needed for successful institutionalised mentorship programs for women scientists in low- and middle-income countries.

Cameroon is a lower-middle-income sub-Saharan African country with a population of over 25 million in 2018.^10^ While 39% of the national population lives below the poverty line, this rate rises to 51.5% for women, with 79.2% of them underemployed.^10^ Although the importance of health research to countries' economic growth has been noted,^11^ the Cameroonian government allocates less than 0.1% of its GDP to health research which, even by African standards, is comparatively low.^12^ According to a UNESCO Institute for Statistics 2019 report, 31.4% of all research produced in Africa has the participation of women, while in Cameroon, the figure is even lower, with only 21.8% of women contributing to research in the country.^3^

### The Mentor Protégée Program (MPP)

**Figure 1:**
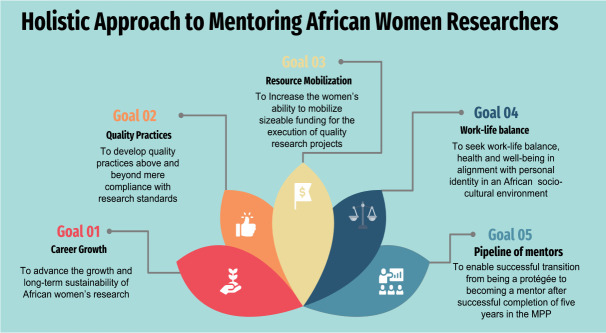
Overview of the HIGHER Women holistic approach to mentoring as a blossoming model with clear goals.

The HIGHER Women MPP's conceptual framework emphasises a holistic approach which is embedded in an integrated structure focusing not only on technical capacity building for health research skills but also providing support for the woman researcher to develop the ability to cope with the African psycho-social environment. The program provides professional guidance to the protégées to facilitate growth and emergence in their careers. The HIGHER Women MPP has five specific objectives or goals of career growth, quality practices, resource mobilisation, work-life balance, and a pipeline of mentors, illustrated in Figure 1 as a blossoming model.

The HIGHER Women MPP has three key components: The Mentor, the Protégée and the Consortium. Each component plays a major role in the program and each bears responsibility for the success of the program. Each Mentor-Protégée relationship is unique and mutually benefits both parties involved. Each of the three components is summarised as follows.

### How the MPP works.

The program matches and pairs mentors with protégées according to the field of expertise, proximity, affiliations and, most importantly, the mentor's ability and willingness to support the protégée's career development and psycho-social needs. The mentor-protégée pair is expected to build a foundation for a successful mentoring relationship with learning, development and support at its core to fulfil clear, mutually defined goals. Although the relationship is based on mutual respect, the protégée is expected to initiate all meetings with the mentor so to strengthen ownership of the partnership. The pair is expected to establish a Mentoring agreement and develop a Mentoring Action Plan that translates goals into easily executable and attainable steps. This plan's progress is evaluated every 12 months by the HIGHER Women consortium.

The mentor-protégées meetings may be held “face to face” virtually, e.g. zoom, WhatsApp, Facebook, google hangouts, skype, etc. or by phone at least four times in a 12-month period. During these meetings, the protégée should be prepared to present her progress toward her professional goals and objectives as per her professional development plan.

Yearly, the protégée must submit a report on her progress by completing the MPP annual report form. Upon acceptance of the annual MPP report, the mentor-protégée is required to re-sign a commitment agreement to renew the commitment to remain in the program and to maintain the relationship.

Every five years, the MPP graduates a group of women from protégée to mentor status. The graduating protégées need to have consistently followed the program and met all criteria for becoming a mentor. This graduation process guarantees a constant pipeline of well-trained potential mentors.

A detailed module for mentor training in implementing the MPP's holistic approach has been documented as part of the collaboration with the Consortium for Mothers, Children, Adolescents and Health Policy and Systems Strengthening (COMCAHPSS) and is available as an open-access guide.^13^

## Methods

The study used a social science single case study design to study the HIGHER Women Cameroon Mentor Protégée Program (MPP). In the social sciences, case study designs are particularly useful for exploring and obtaining insights into complex processes and phenomena within their real-life context. Data sources are a desk review of program documents and two cross-sectional surveys (2018 and 2022, respectively) of mentors and protégées.

### Data Collection Methods for evaluation

A first survey focused on measurement and assessment of the mentoring program processes was administered between 1 and 30 July 2018 using an online structured questionnaire using survey monkey and sent to all 103 HIGHER Women registered protégées. No mentors participated in the 2018 survey. A total of 35 protégées responded to the online survey giving a 34% response rate. Key informant in-depth interviews were conducted face-to-face with 20 purposively selected based on willingness to participate protégées and mentors.

The questionnaire was adapted from the University of North Carolina School of Medicine Evaluation of Mentoring Best Practices tool.^14^ It included questions on demographics, number of contacts per year between mentor and protégée, mentor's accessibility, the level of satisfaction with support received from the mentor, the motivation brought on by MPP's support, positive and negative aspects of experience with the MPP and suggestions for improvement.

In 2022, the same 103 protégées surveyed in 2018 were sent a second online survey using survey monkey. The second survey focused more on assessing early measures of goal attainment, specifically goal 1, career growth. Goal 2, is quality practices, and Goal 3 is resource mobilisation. The 10-question survey was developed inspired by a previously validated survey on mentorship evaluation.^15^ The questions aimed to gauge changes in professional output, more specifically on the number of peer-reviewed articles before and after joining HIGHER women, on the number of national and international conferences attended before and after joining HIGHER women, and on promotions after joining HIGHER women. Each HIGHER Women protégée registered received a unique link to the online questionnaire on April 1st, 2022. The questionnaire remained open until April 5, 2022. Reminders to fill out the survey were sent numerous times daily on the consortium's WhatsApp group while the survey was open. Among the 103 eligible protégées that received the link, 77 completed the survey yielding an overall response rate of 74.7%. Once 2022 data collection was complete, all information linking individual participants to their survey responses was destroyed, creating a completely anonymous dataset.

### Data analysis

Analysis was done with Excel (version 16.56 2021) to generate descriptive analyses such as frequencies and percentages. Apart from simple frequencies and cross tables,

Pearson correlations were performed to test the strength and direction of linear relationships between variables. All statistical tests were two-sided with a significance level of < 0.05. The qualitative data from the face-to-face interviews were analysed manually using thematic content analysis.

## Results

### 2018 Online Survey and KI interview findings

**Figure 2:**
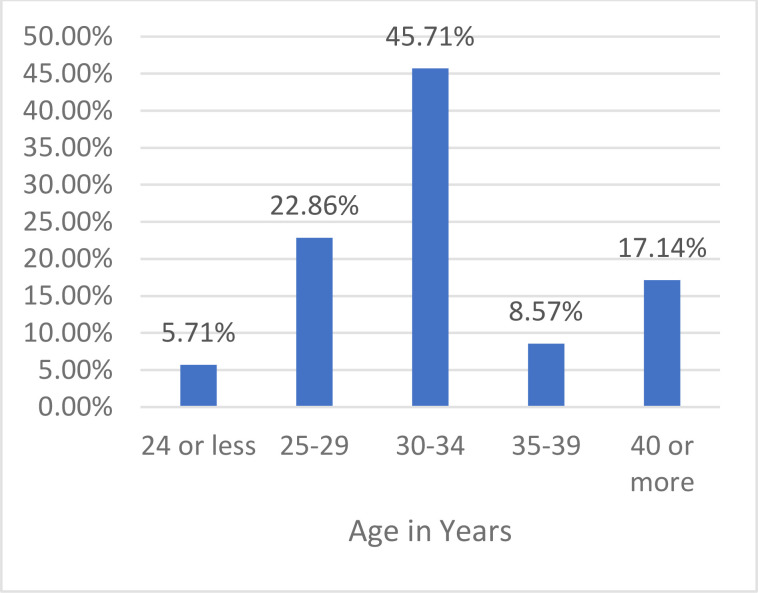
Distribution of 35 Protégées by age in the 2018 survey

**Figure 5:**
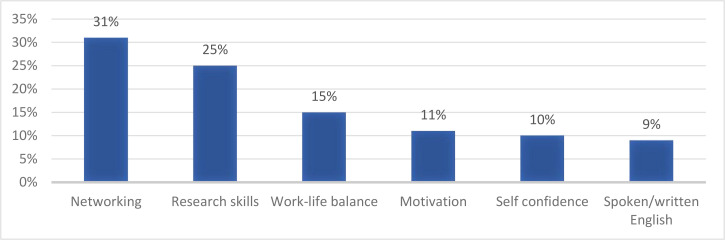
Skills and competencies improved after five years as an MPP protegee: 2018 survey

Figure 2 summarises the ages distribution of the 35 protégées responding to the 2018 survey, and Figure 5, the skills and competencies that the protégées cited as most improved during their participation in the program. Areas in which the protégées felt that the MPP could do better included strengthening their fundraising skills (38%), stronger mentor-protégée relationship (26%) and collaboration with other local research institutions (21%).

The majority of Protégées (54.29%) had a Master's degree, 40% a PhD, while 5.71% identified as Post-doctoral researchers. About 56% of Protégées worked and lived in Yaoundé, the capital of Cameroon in the Center region; while 18% worked or studied in Buéa, the South-West region. Other locations of Protégées were Douala (12%), Bamenda in the North-West region (9%) and Bangangté in the West region (6%). The main areas of research were Biochemistry (25%), Public Health (18%), Parasitology (11%), Microbiology (11%) and Clinical Biology, Pharmacy, Psychology, and Infectious Diseases (32%).

**Figure 3:**
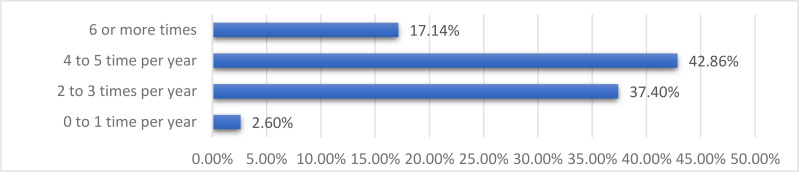
2018 Survey showing the frequency of contact between mentor and protegee

**Figure 4:**
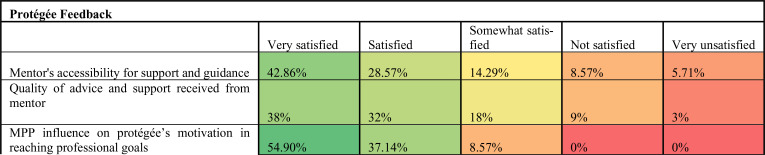
Protegee's satisfaction on various MPP functions, 2018 survey

Figure 3 summarises the frequency of contact between mentor and protegees. Major benefits from participating in the HIGHER Women MPP were reported. Figures 4 and 5 show the areas of satisfaction and improvements in skills and competencies during the five years of the programme.

Examples of the kinds of these benefits are summarised below using quotations from the interview transcripts.

*“Since I joined, the HIGHER Women consortium has greatly impacted my life. HIGHER gives me a network of researchers that I can count on. For example, I contacted a mentor in Centre Pasteur when I needed some microorganisms, and she made herself available and directed me on how to proceed on obtaining the materials”*

### Protégée’ 2018 KI interview.

“HIGHER Women has helped me to see the need to go for a PhD program. It has also helped me build confidence in myself as a health researcher.” Protégée’ 2018 KI interview

*“I appreciate the elder/younger woman relationship with my Mentor. The motivation from the achievements of other HIGHER women, the announcements sent by WhatsApp and in our email boxes, the celebration of family events, the feeling of family we get from each other and specially the joy of togetherness”* Protégée’ 2018 KI interview

*“Since I became a HIGHER Woman, I have learned to have more confidence in myself, I better manage my time and I organize my work and my life. I have managed to submit abstracts that was selected in two international conferences. Before joining the consortium, I never seemed to find time for writing.*” Protégée’ 2018 KI interview

*“The motivation and moral support I get from the Mentors and other HIGHER Women is very good and it makes me venture for things I was not able to do before. I also appreciate the fact that I can approach another mentor who might not be my direct mentor, if my mentor is unavailable. I am proud to belong to an association that strives to improve and encourage women to be their best. In fact, I shared this experience in a conference I attended in Japan and this idea was highly appreciated.”* Protégée’ 2018 KI interview

The evaluation also revealed that, since joining the program, the HIGHER women protégées reported the following accomplishments: 1) major research grants secured, resulting HIGHER Women events and active mentoring, 2) a significant increase in scientific publications in peer-review journals by protégées 3) more protégées presenting their research findings in national and international conferences as well as 4) more protégées rising in academic ranks in universities and research institutions.

### 2022 Online Survey findings

Table 1 summarises the protégée number of years of experience within the HIGHER Women consortium reported in 2022 and the average number of peer-reviewed articles published per protégée.

**Table 1:** Average number of peer-reviewed articles published against the number of years as a HIGHER women protégée

Number of years as HIGHER Woman protégée	The average number of articles published as a HIGHER Woman protégée
**Up to 1 year**	3
**Up to 2 years**	0
**Up to 3 years**	1
**Up to 4 years**	4

A Pearson correlation was performed to test whether there was an association between the number of peer-reviewed articles published and the number of years of experience as a HIGHER Women protégée. The r of 0.41, although not statistically significant (p = .592), suggests a positive correlation between the increased number of peer-reviewed articles and the increased number of years as a HIGHER Women protégée. . The consortium's achievements since its inception are shown in Table 2 by the five goals in the blossoming model.

**Table 2:** Summary of progress towards the five HIGHER Women Goals of holistic mentoring

Goals		Achievements
**Goal 1 – Career** **Growth**	“To advance the growth and long-term sustainability of African women's research”	By 2022, over 150 early -career women researchers have benefited from the HIGHER Women MPP. A mentoring guide was developed with the support of Consortium for Mothers, Children, Adolescents and Health Policy and Systems Strengthening (COMCAHPSS). The consortium has published 4 articles in high profile journals. 28.57% protégées report having collaborated in scientific projects with other HIGHER Women members 7.79% protégées report co-authoring more than 4 scientific articles with other HIGHER women members since 2015
**Goal 2 – Quality Practices**	“To develop quality practices above and beyond mere compliance with research standards”	Good Clinical Practices seminars were organized in 2016 and 2018
**Goal 3 – Resource mobilization**	“To increase the women's ability to mobilize sizeable funding for the execution of quality research projects.”	The Consortium received financial support from: World Health Organization's Special Programme for Research and Training in Tropical Diseases (WHO/TDR) Canada's International Development Research Centre (IDRC Canada) The Dangote Foundation Cameroon's Ministry of Women Empowerment and the Family Cameroon's Ministry of Higher Education In July 2016, a 3-day proposal writing retreat was attended by 68 HIGHER Women members
**Goal 4 – Work-life balance**	“To seek work-life balance, health and well-being in alignment with personal identity in an African socio-cultural environment”	The 2019 annual forum heavily focused on the family, society, and career. During the annual fora, “fireside conversations” where mentors and protégées exchanged their experiences and professional journeys including tips on handling societal and cultural challenges on their careers.
**Goal 5- Pipeline of** **mentors**	“To enable successful transition from being a protégée to becoming a mentor after successful completion of five years in the MPP”	In 2019, seven (7) women graduated from protégée to mentor sta-tus. They have been attributed a total of 21 protégées

## Discussion

The HIGHER Women consortium was born out of a dire need to support women researchers in Cameroon through mentoring. The formal and well-structured MPP evolved quickly to become a pillar for women embarking on a career in research for health in Cameroon. The holistic approach (Figure 1) to mentoring adopted by the consortium not only accentuates the technical and economic aspects of women's careers in research, but a particular emphasis is also placed on optimising the intrinsic characteristics of an African woman to allow the researcher to wholly flourish in her personal life as well as in her career. Social roles such as mother, wife, housekeeper, family member (daughter, sister, child), community member, and friend weigh heavily on African women as for other women around the world. In a study conducted in South Africa on the phenomenon of work-life balance for professional women, it was found that having strong support systems positively influences success and performance while enhancing her physical and mental well-being.^16^

Woman researchers are called to wear numerous hats at work, in the community and at home. On the premise that a woman's career will better grow on the foundation of a fulfilled personal life, the HIGHER Women consortium's MPP endeavours to train women in achieving work-life balance, health, and well-being in alignment with personal identity and socio-cultural environment.

It is important to take stock and identify lessons from the experience of this mentoring program for women in health research in Cameroon, as this special article has tried to do. Mentoring and other capacity-building programs usually demonstrate outcomes in the medium to long term. The first survey and key informant interviews in 2018 generated information about processes.

The assumption was that these processes would lead to the desired goals in the medium to long term. The 2nd survey conducted in 2022, five years later, enabled the team to begin to assess medium-term progress towards achieving the goals of career growth, quality practices, resource mobilisation, work-life balance and pipeline of mentors depicted in the blossoming model. The results suggest that over time desired results are beginning to emerge and increase the longer the protégées were mentored as HIGHER Women. Our results are corroborated by several studies of mentoring women. In a study conducted in low- and middle-income countries, it was found that mentoring early career women in Health Policy and Systems Research strengthened their skills in producing strong manuscripts.^17^ In a similar study conducted with women in post-doc programs in middle and low- and middle-income countries, it was also found that early-career women prospered when provided with the necessary space for learning, reflection, and mentorship.^18^

In the past few decades, the African continent has seen an improvement in investments in health research. Although these investments are needed and appreciated, they are insufficient since the continent's research output continues to lag significantly behind the rest of the world.^19^ The HIGHER women MPP addresses this issue by providing tools and imparting skills meant to advocate for additional investments in women's health researchers. Ultimately, the consortium's goal is to add to the critical mass of well-trained and skilled African researchers capable of conducting African-led research in Africa to improve our communities' health and the continent's transformation. Africa is known for its hyper-dependence on foreign aid in various sectors, including health research.^20^ The MPP protégées, as they are initiating their research careers are made conscious of the need to battle against this dependence.

The MPP's mentoring approach facilitates its sustainability in the long term. The mentoring process starts with a meticulously matched mentor-protégée duo regarding their mutual career paths, personal and professional experiences, and motivation to participate in the program. Although the basic mentoring relationship is between the protégée and the mentor, the consortium is an ever-present and integral element that serves as the program's backbone. The protégées' interviews have revealed the consortium's activities not only attracted them to MPP but also encouraged them to continue to actively participate in the program. Mentoring programs have come and gone in Africa and in other parts of the world. The HIGHER Women consortium perpetuates itself by continuously building a pipeline of mentors and by enabling a successful transition from protégée to mentor after the successful completion of five years in the MPP. Since its inception in 2015, the MPP has graduated nine additional mentors, which expands the network and allows for a wider reach in the recruitment of new protégées.

In this paper, we aimed to present the consortium, the MPP program and show its effect on early-career women in health research in Cameroon. There are limitations. The evaluation that was conducted in 2018 was limited by the minimal response rate. As a mitigation measure, in 2022, a new survey was conducted with the same HIGHER Women and this time we achieved a 77% response rate that permits more confidence in our findings. As well, the 2022 survey could have benefitted from a longer duration for data collection. Based on our findings, we believe that additional research to track progress over time across all five objectives in the blossoming model is needed to generate more insights on what works, challenges and opportunities for using mentoring as an intervention to support career development for women scientists in health and to bridge some of the gender inequity gaps.

Numerous mentoring programs in Africa and around the world have met some challenges, such as sustainability, stunted expansion, inhibited mentee retention, reduced mentor motivation, lack of institutional support as well as the devasting global impact of COVID-19. To become and remain successful and achieve excellent results, mentoring programs have to adapt to and address these challenges.

Lessons from the HIGHER Women mentoring approach for the development and sustainability of similar programs and for the continuous improvement of the program itself include the following. The structured HIGHER women mentoring approach is of potential benefit to other countries in Sub-Saharan Africa seeking to increase the participation of women in science as researchers and reduce the current wide gender gaps. National and international funders should consider investing in supporting such approaches.

Programs such as the HIGHER Women's MPP require financial resources, not only for their development and implementation but also for their sustainability. Coordination of the effort requires at least one officer in key research institutions dedicated to ensuring the program's functioning and its day-to-day activities. Resources are needed to facilitate the development of mentoring skills as well as to organise capacity-building events to enliven the program and maintain the members' enthusiasm. The experience of communication between mentors and protegées using various online mechanisms shows that establishing and developing online platforms and approaches can be an effective way to broaden the program's reach so that participants are not limited by geography or by time.

## Conclusion

The field of health research in Africa is on the rise with many emerging opportunities and women have an important role to play. However, it must be facilitated and mentoring programs such as the one described in this special article are part of the way forward. Documenting the approaches and lessons in this special article is an important step forward to making the innovations and lessons available to support emerging women researchers in low- and middle-income countries.

## References

[1] Roca A, Okomo U, Usuf E, Oriero EC, Janha R, Achan J (2018). African women working in global health: closing the gender gap in Africa?. Lancet Glob Health.

[2] Kwedi Nolna SK, Essama Mekongo PE, Leke RGF (2017). Mentoring for early-career women in health research: The HIGHER Women Consortium approach. Vol. 2, Global Health, Epidemiology and Genomics. 2017. Glob Health Epidemiol Genom.

[3] UNESCO Institute for Statistics (2019). Women in Science.

[4] Nearing KA, Nuechterlein BM, Tan S, Zerzan JT, Libby AM, Austin GL (2020). Training Mentor-Mentee Pairs to Build a Robust Culture for Mentorship and a Pipeline of Clinical and Translational Researchers: The Colorado Mentoring Training Program. Acad Med.

[5] Sorkness CA, Pfund C, Ofili EO, Okuyemi KS, Vishwanatha JK, Zavala ME (2017). A new approach to mentoring for research careers: The National Research Mentoring Network. BMC Proceedings.

[6] Diggs-Andrews KA, Mayer DCG, Riggs B (2021). Introduction to effective mentorship for early-career research scientists. BMC Proceedings.

[7] Essence on Health Research (2020). Health research capacity strengthening in low and middle-income countries: current situation and opportunities to leverage data for better coordination and greater impact. Mechanism for the review of investments in health research capacity strengthening in LMICs.

[8] Mekongo PE, Nolna SK, Ngounoue MD, Ndongo JT, Ndje MN, Nguefeu CN (2018). The Mentor-Protégée Program in health research in Cameroon. Lancet.

[9] de Janasz SC, Godshalk VM (2013). The Role of E-Mentoring in Protégées' Learning and Satisfaction. Group & Organization Management.

[10] The World Bank (2021). The World Bank in Cameroon.

[11] Guthrie S, Cochrane G, Deshpande A, Macaluso B, Larivière V (2019). Understanding the contribution of UK public health research to clinical guidelines: A bibliometric analysis. F1000Re.

[12] Tonjock R (2020). Current statistics in Science, Technology and Innovation in higher education in Cameroon and the establishment of gender participation. Afr Journal of Rural Dev.

[13] The HIGHER Women Consortium (2018). Effective strategies for quality mentoring of health researchers.

[14] University of North Carolina School of Medicine (2017). Evaluation of Mentoring Best Practices UNC School of Medicine).

[15] Yukawa M, Gansky SA, O'Sullivan P, Teherani A, Feldman MD (2020). A new Mentor Evaluation Tool: Evidence of validity. PLoS One.

[16] Whitehead T, Kotze ME (2003). Career And Life-Balance Of Professional Women: A South African Study. SA Journal of Human Resource Management.

[17] Kwamie A, Jalaghonia N (2020). Supporting early-career mentorship for women in Health Policy and Systems Research: A vital input to building the field. Health Policy and Planning.

[18] Lembani M, Teddy G, Molosiwa D, Hwabamungu B (2016). Post-doctoral research fellowship as a health policy and systems research capacity development intervention: A case of the CHESAI initiative. Health Research Policy and Systems.

[19] Kasprowicz VO, Chopera D, Waddilove KD, Brockman MA, Gilmour J, Hunter E (2020). African-led health research and capacity building- is it working?. BMC Public Health.

[20] Laabes EP, Desai R, Zawedde SM, Glew RH (2011). How much longer will Africa have to depend on western nations for support of its capacity-building efforts for biomedical research?. Tropical Medicine and International Health.

[21] Konyeha S, Agwam G, Musa E, Ngonadi IV, Afehomo AC (2021). Initiatives and role of women scientist forums with mentorship opportunities in STEM. African Journal of Health, Safety and Environment.

[22] Ntoumi F (2019). Supporting female scientists in Africa. Lancet.

[23] Bilesanmi-Awoderu JB (2006). Enhancing Female Participation And Achievement In Science And Technology Using Mentoring Technique: Perceptions Of Nigerian Female Scientists And Engineers. African Journal of Cross-Cultural psychology and sport facilitation.

